# Therapeutic Effects of Apremilast on Enthesitis and Dactylitis in Real Clinical Setting: An Italian Multicenter Study

**DOI:** 10.3390/jcm12123892

**Published:** 2023-06-07

**Authors:** Alberto Lo Gullo, Andrea Becciolini, Simone Parisi, Patrizia Del Medico, Antonella Farina, Elisa Visalli, Ylenia Dal Bosco, Aldo Biagio Molica Colella, Federica Lumetti, Rosalba Caccavale, Palma Scolieri, Romina Andracco, Francesco Girelli, Elena Bravi, Matteo Colina, Alessandro Volpe, Aurora Ianniello, Maria Chiara Ditto, Valeria Nucera, Veronica Franchina, Ilaria Platé, Eleonora Di Donato, Giorgio Amato, Carlo Salvarani, Simone Bernardi, Gianluca Lucchini, Francesco De Lucia, Francesco Molica Colella, Daniele Santilli, Natalia Mansueto, Giulio Ferrero, Antonio Marchetta, Eugenio Arrigoni, Rosario Foti, Gilda Sandri, Vincenzo Bruzzese, Marino Paroli, Enrico Fusaro, Alarico Ariani

**Affiliations:** 1 Unit of Rheumatology, Department of Medicine, ARNAS “Garibaldi”, 95124 Catania, Italy; 2 Internal Medicine and Rheumatology, Department of Medicine, Azienda Ospedaliera Di Parma, 43126 Parma, Italy; beccio@yahoo.it (A.B.);; 3 Department of General and Specialistic Medicine, Azienda Ospedaliero-Universitaria Città della Salute e della Scienza di Torino, 10126 Torino, Italy; 4 Internal Medicine Unit, Civitanova Marche Hospital, 62012 Civitanova Marche, Italy; 5 Internal Medicine Unit, Ospedale Augusto Murri—Fermo, 63900 Fermo, Italy; 6 Unit of Rheumatology, Ospedale San Marco, 95121 Catania, Italy; elivisa21@gmail.com (E.V.); yleniadalbosco@gmail.com (Y.D.B.);; 7 Unit of Rheumatology, Ospedale Papardo, 98158 Messina, Italy; aldomolica@alice.it (A.B.M.C.);; 8 Unit of Rheumatology, Azienda Unità Sanitaria Locale di Modena, 41121 Modena, Italy; fedelumetti@gmail.com; 9 Department of Medical-Surgical Sciences and Biotechnologies, Sapienza University of Rome, 00185 Roma, Italy; rosalba_caccavale@yahoo.it (R.C.); marino.paroli@uniroma1.it (M.P.); 10 Unit of Internal Medicine and Rheumatology, ASL Roma 1—Presidio Nuovo Regina Margherita, 00153 Roma, Italy; palma.scolieri@gmail.com (P.S.);; 11 Asl1 Imperiese, 18100 Sanremo, Italy; 12 Internal Medicine Unit, Ospedale “Morgagni–Pierantoni” di Forlì, 47121 Forlì, Italy; 13 Department of Rheumatology, Ospedale “Guglielmo da Saliceto”, 29121 Piacenza, Italy; e.bravi@ausl.pc.it (E.B.);; 14 Department of Biomedical and Neuromotor Sciences, Alma Mater Studiorum—Università di Bologna, 40136 Bologna, Italy; 15 Rheumatology Unit, Ospedale Sacro Cuore Don Calabria, 37024 Negrar di Valpolicella, Italyantonio.marchetta@sacrocuore.it (A.M.); 16 SL NOVARA, 28100 Novara, Italy; 17 Rheumatology Unit, University of Modena and Reggio Emilia, 41125 Modena, Italy

**Keywords:** psoriatic arthritis, apremilast, enthesitis, dactylitis

## Abstract

Introduction: Enthesitis and dactylitis are difficult-to-treat features of psoriatic arthritis (PsA), leading to disability and affecting quality of life. Objective: The aim of this study is to evaluate enthesitis (using the Leed enthesitis index (LEI)) and dactylitis at 6 and 12 months in patients treated with apremilast. Methods: Patients affected by PsA from fifteen Italian rheumatological referral centers were screened. The inclusion criteria were: (a) enthesitis or dactylitisphenotype; (b) treatment with apremilast 30 mg bid. Clinical and treatment history, including PsA disease activity, were recorded. Mann–Whitney and chi-squared tests were used to assess the differences between independent groups, and Wilcoxon matched pairs signed-rank test assessed the differences between dependent samples. A *p*-value of <0.05 was considered statistically significant. Results: The Eph cohort consisted of 118 patients (median LEI 3); the Dph cohort included 96 patients with a median dactylitis of 1 (IQR 1–2). According to an intention to treat analysis, 25% and 34% of patients with enthesitis achieved remission (i.e., LEI = 0) in T1 and T2. The remission of dactylitis was 47% in T1 and 44% in T2. The per protocol analysis (patients observed for at least 12 months) showed that both dactylitis and LEI significantly improved in T1 (median LEI 1 (IQR 1–3)) and T2 (median LEI 0 (IQR 1–2)). Conclusion: Eph and Dph PsA patients treated with apremilast experienced a significant improvement in enthesitis and dactylitis activity. After 1 year, enthesitis and dactylitis remission was achieved in more than one-third of patients.

## 1. Introduction

Psoriatic arthritis (PsA) is a chronic inflammatory disease that affects up to 30% of patients with psoriasis. PsA may affect peripheral and sacroiliac joints, and the spine [1,2]. In addition to synovitis, musculoskeletal manifestations may present as enthesitis and dactylitis [1,2,3,4], and are usually associated with more severe disease [5,6,7]. Enthesitis and dactylitis often occur in the lower limbs and can cause tenderness or pain when standing and walking [8,9] or can alter motor function by limiting one’s ability to hold objects [10]. Both those conditions are associated with impaired function, and they have a negative impact on quality of life [3,4,8,9]. Dactylitis and enthesitis are hallmark features of PsA that may be associated with more serious diseases [2].

Dactylitis is one of the distinguishing features of spondyloarthropathies, particularly of PsA. In the Toronto cohort of psoriatic arthritis patients, this was reported as a common feature, occurring in 48% of patients. Dactylitis was most often observed at the first medical visit (69%), likely because dactylitis is a well-recognized and distinguishing feature that triggers referral to a specialist clinic [9]. On the other hand, identifying enthesitis in patients with PsA can be challenging. Enthesitis can be asymptomatic or can mimic symptoms related to other disorders, such as mechanical injury and tendinitis [4]. Although different indices have been evaluated to measure enthesitis, many of themwer e developed specifically for patients with AS [4]. The Leed Enthesitis index (LEI) correlates with the clinical parameters of disease activity in patients with PsA and is easy to perform. It provides information on the activity of the disease and helps to monitor treatment response [4].

Therapeutic strategies for PsA include systemic therapy with conventional disease-modifying antirheumatic drugs (DMARDs) and biological agents. However, to date, evidence of the effectiveness of conventional DMARDs in PsA enthesitis or dactylitis has not yet been assessed [11]. Apremilast is an oral inhibitor of the phosphodiesterase 4 that EU-LAR guidelines recommend using in PsA patients with relatively mild disease or when other agents are contraindicated [12]. The PALACE 1, 2, and 3 studies demonstrated the efficacy and safety of apremilast in patients with active PsA despite prior conventional DMARDs and/or biological therapy. Data from the PALACE studies allowed for a comprehensive analysis of different aspects, including treatment outcomes [12,13,14,15]. However, to date, real clinical data on the use of apremilast for the treatment of PsA enthesitis and dactylitis are limited.

The main aim of this Italian large multicenter observational retrospective analysis is to report the effects on LEI and Dactylitis at 6 and 12 months in patients being treated with apremilast.

## 2. Materials and Methods

### 2.1. Ethics

As a part of the BIRRA (BIologics Retention Rate Assessment) project, this retrospective study was designed to evaluate the efficacy of apremilast on enthesitis and dactylitis. The study was performed according to the Declaration of Helsinki principles, and it was approved by the local Ethics Committees (the main is the Comitato Etico dell’Area Vasta Emilia Nord, protocol code 34,713, approved on 28 August 2019).

### 2.2. Patients

The analyzed population is part of the BIRRA (BIologics Retention Rate Assessment) project, an observational retrospective study [16]. All PsA consecutive patients from fifteen Italian rheumatological referral centers were included if they fulfilled the following criteria: (a) enthesitis (Eph) or dactylitis (Dph) phenotype; (b) treatment with apremilast 30 mg bid. Patients who received apremilast and bDMARDs at the same time or only for dermatologic indication (i.e., psoriasis (PsO)) were excluded.

### 2.3. Data

For each patient, the following data were recorded: general characteristics (age, sex, body mass index (BMI), smoking habit, PsA and PsO onset, and date of diagnosis), PsA phenotype (enthesitic and dactylics subtype), apremilast-related information (date of the first and last treatment administration), other PsA treatment history (both csDMARDs and bDMARDs), PsA disease activity (number of tender/swollen joints, painful enthesis and fingers affected by dactylitis, C-reactive protein, pain Visual Analog Scale, and patient global assessment values), and the presence of comorbidities at baseline, 6 months (T1), and 12 months (T12). DAPSA, LEI, and Dactylitis were assessed as measures of disease activity [17]. Enthesitis was determined by the presence or absence of tenderness at the following sites of the LEI: lateral epicondyle (left and right), medial femoral condyle (left and right), and Achilles tendon insertion (left and right) [18]. The absence (score = 0) or presence (score = 1) at each of the six sites was evaluated and added to produce an LEI total score ranging from 0 to 6. Dactylitis is defined as “uniform swelling such that the soft tissues between the metacarpophalangeal and proximal interphalangeal, proximal and distal interphalangeal, and/or distal interphalangeal joint and digital tuft are diffusely swollen to the extent that the actual joint swelling could no longer be independently recognized” [3,17]. Dactylitis score was evaluated in patients with a dactylitis count > 0 at baseline, reflecting the presence (score = 1) or absence (score = 0) of dactylitis in each of the 20 digits (the possible dactylitis count ranges from 0 to 20) [19,20]. Cancer, HBV, HCV, latent tuberculosis (TB), and other chronic infections were considered as relevant comorbidities.

### 2.4. Statistical Analysis

Continuous variables were reported as median value and interquartile range (IQR); categorical values were reported as a percentage. The effect of apremilast on enthesitis and dactylitis was evaluated both with an “intention to treat” and “per protocol” analysis. The Wilcoxon matched pairs signed-rank test assessed the differences between basal, T1, and T2 of the above-mentioned scores. Remission was expressed as a percentage of patients achieving Dactylitis score or LEI equal to 0 on T1 or T2.

Logistic regressions verified correlation between a Dactylitis score or LEI equal to 0 in T2 and the following factors: age, sex, BMI, smoking habit, relevant comorbidity, PsA disease duration, baseline Dactylitis score or LEI as appropriate, and concomitant csDMARDs treatment. We performed univariate logistic regression analysis on all variables and included those with a *p*-value < 0.1 into multivariate logistic regression analysis to determine independent prognostic factors of Dactylitis score or LEI equal to 0 in T2.

A *p*-value < 0.05 was considered statistically significant. Statistical analysis was performed using an online application (www.statskingdom.com, last visit 20 December 2022).

## 3. Results

### 3.1. Patient Characteristics

Patients with Eph and Dph at baseline were 118 and 96, respectively. The patients with enthesitis and dactylitis activity reported in T0, T1, and T2 were 66 and 64, respectively. The baseline characteristics of both groups are in Table 1 and Supplementary Table S1. Forty-seven patients had synchronous dactylitis and enthesitis.

### 3.2. Efficacy Results

**Figure 1:**
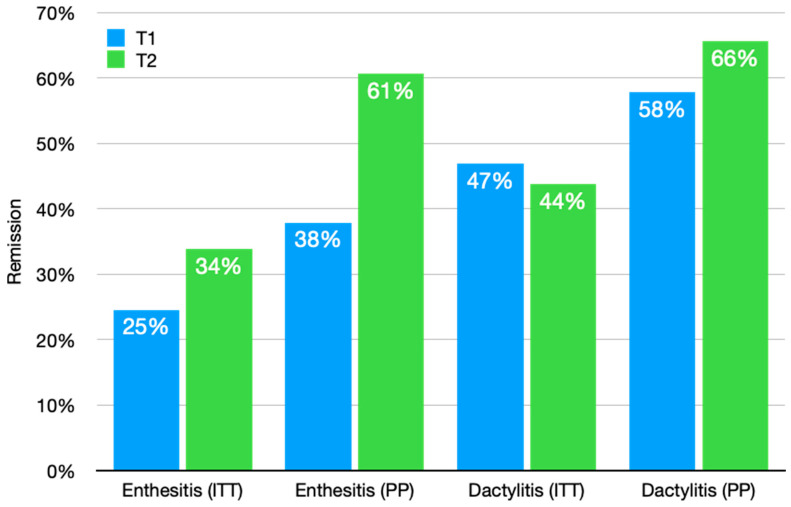
Percentage of patients achieving enthesitis or dactylitis remission after six (blue) and twelve (green) months. All values are provided according to intention to treat (ITT) and per protocol (PP) analysis.

**Figure 2:**
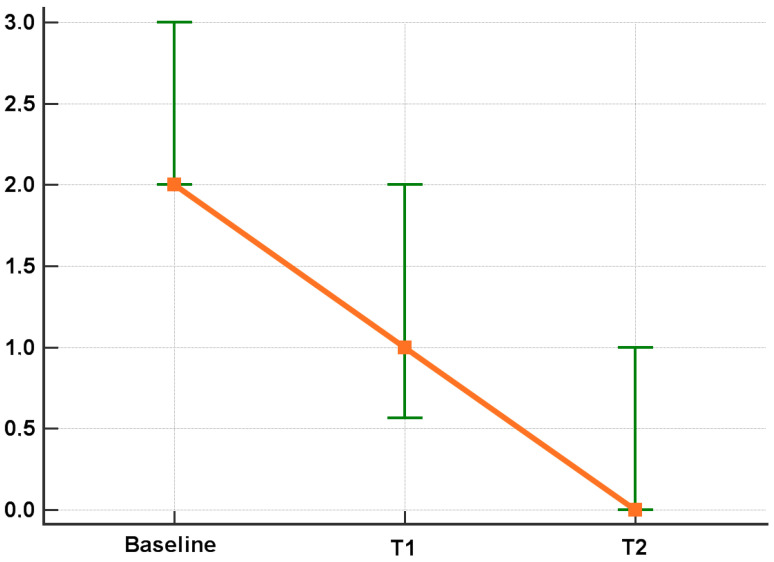
LEI score after six and twelve months of apremilast treatment. All values are provided according to the per protocol (PP) analysis. IQR in green, orange squares refer to median values.

**Figure 3:**
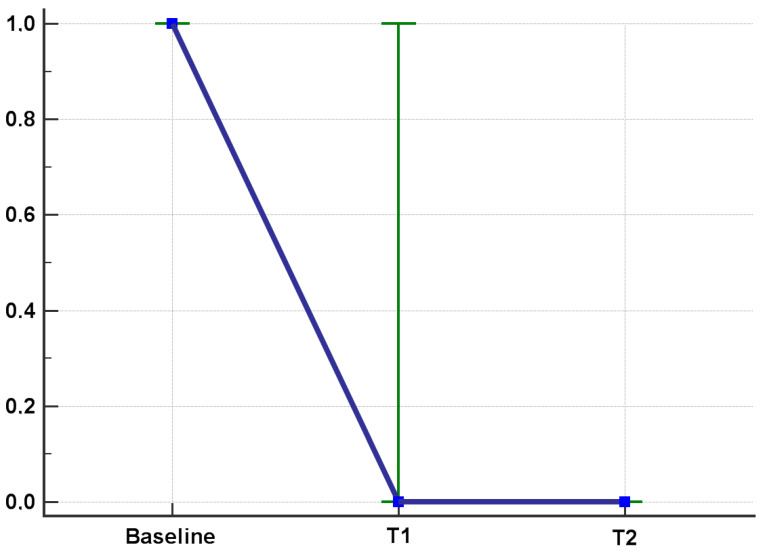
Dactylitis score after six and twelve months of apremilast treatment. All values are provided according to the per protocol (PP) analysis. IQR in green, blue squares refer to median values.

According to the intention to treat analysis, the Eph patients that achieved remission (i.e., LEI = 0) in T1 and T2 were 25% and 34%, respectively. In the Dph group, 47% and 44% of patients experienced remission after 6 months and 12 months (Figure 1). The per protocol analysis showed a LEI improvement in T1 and T2 (*p* < 0.001 for both T1 vs. T0, T2 vs. T1) (Figure 2). Similarly, the Dactylitis score decreased to 0 both in T1 and T2 (*p* < 0.001 for both T1 vs. T0, T2 vs. T1) (Figure 3).

### 3.3. Predictors of Remission

Logistic regression showed that the baseline LEI and Dactylitis score are predictive of remission. Moreover, dactylitis remission is associated with the patient’s age and csDMARDs concomitant use. Table 2 and Table 3 report the univariate and multivariate logistic regressions of variables associated with the achievement of remission of enthesitis and dactylitis at T2.

## 4. Discussion

The main results of this study are that the resolution of enthesitis and dactylitis was observed in 34% and 44% of patients after one year of treatment, demonstrating the efficacy of apremilast in these conditions. Moreover, it seems that the achievement of remission is associated with dactylitis and LEI scores assessed at the beginning of treatment.

PsA is a multifaceted disease, with enthesitis and dactylitis as the first manifestations for many patients. Both enthesitis and dactylitis require considerable therapeutic effort and can lead to disability or compromise quality of life. Furthermore, these conditions also contribute to an exaggeration of the perceived disease burden in PsA [3,5,10,18]. Dactylitis, in general, has a more serious disease phenotype that is independently associated with greater swollen joint count, CRP, synovitis, and bone erosions in PsA [21]. The current treatment strategy for PsA is systemic therapy with DMARDs and biological agents; indeed, evidence suggesting that conventional DMARDs are effective in treating enthesitis or dactylitis is scarce [11]. In the SEAM-PsA trial, the resolution of enthesitis was reported in 43.1%, 52.6%, and 47.5% of patients on methotrexate monotherapy, etanercept monotherapy, and combination therapy, respectively, with no difference between the three patient groups [22]. The results of pooled analysis of patients with enthesitis and/or dactylitis at baseline in the PALACE studies demonstrated that apremilast is a valid option to reduce the severity of both symptoms in patients with PsA treated with apremilast for three years [12,13,14,15,16,23]. The studies mentioned above do not replicate a real-world setting. In fact, patients with severe comorbidities (i.e., cancer, chronic infection, etc.), with less than three tender and swollen joints, and who were on treatment with more than two csDMARDs, were not included in the trials. Most of the studies in real setting are focused on patients with only psoriasis, and the specific data regarding dactylitis and enthesitis are missing. Abignano et al., in the first real-life report of apremilast use in PsA, reported that 38 (60%) of a total of 71 PsA patients had enthesitis and dactylitis at baseline; 60.8% had a response to apremilast but the exact percentage of resolved dactylitis and enthesitis was not reported [24]. More recently, an observational study from Belgium on a small number of Eph and Dph patients (24 and 21) treated with apremilast reported that the 6 months remission of dactylits and enthesitis was achieved in 71% and 37.5%, respectively [19]. The data from the Belgium study are different from our study because the authors reported a greater remission rate of both enthesitis and dactylitis, and the median BMI was higher than our population. Approximately 35% of Dph patients and 28% of Eph patients received prior csDMARD treatment in our study, whereas data on prior treatments in patients with enthesitis and dactylitis alone cannot be extrapolated from the Belgian study; however, most of the Belgian psoriatic arthritis patients had received treatment before using apremilast. In the RAPPER study from Italy, a mean LEI of 2 ± 1.4 was reported in 35 patients with PsA at baseline. After 6 months of treatment with apremilast, the results were reported in only 6 patients (median LEI of 0.2 ± 0.8); therefore it is difficult to draw clear conclusions about efficacy apremilast on the LEI based on these reports [25]. Moreover, the aforementioned study enrolled patients with a long disease duration and all included subjects were from tertiary rheumatology centers, potentially limiting the reproducibility of data to the whole Italian population with PsA. In a recent real-life study from Greece that had similar characteristics to our population 30.7% (50/163) of patients had enthesitis at baseline (LEI > 0) and 12.4% (20/161) had dactylitis (Dactylitis Severity Score > 0). Patients treated with apremilast achieved complete resolution of enthesitis in 64.7% (22/34) and 83.3% of cases (25/30) after 24 and 52 weeks of treatment, respectively. Instead, a complete resolution of dactylitis was reported in 72.7% (8/11) and 90.0% (9/10) of patients at 24 and 52 weeks, respectively [26].

The Eph and Dph patients were selected from a more numerous PsA real-life cohort [16] and they represented 31% and 34% of that cohort. These results are consistent with other published studies that reported enthesitis in a range of 34–83% and dactylitis in about 32–48% of patients with PsA [3,5,7,9]. Furthermore, data from our cohort indicate that patients had mild disease activity at baseline as evidenced by a moderate DAPSA in both groups, which is consistent with the EULAR guidelines that recommend treatment with apremilast in patients with mild disease or if other agents are contraindicated [27].

The results from this study showed that apremilast is effective in reducing the severity of both enthesitis and dactylitis. By month 6, statistically significant improvements in enthesitis and dactylitis were already observed, with further improvement at 12 months in both groups. Our results are different from the pooled PALACE studies, in which remission of dactylitis and enthesitis was reached in higher percentage, but after 3 years. In fact, in the pooled analysis of PALACE studies, at week 156, 55% of patients treated with apremilast 30 mg bid and 55.1% treated with apremilast 20 mg bid achieved a Maastricht Ankylosing Spondylitis Enthesitis Score (MASES) of 0. Regarding dactylitis in the same analysis at week 156, 79.6% of patients treated with apremilast 30 mg bid and 73.9% treated with apremilast 20 mg bid achieved a dactylitis count of 0 [23]. On the other hand, in patients with PsA on treatment with MTX combined with continuous NSAIDs, a significant improvement in acute dactylitis, and LDI basic was achieved in 9 months. Similarly, LEI significantly improved, and 63.01% of patients reached Minimal Disease Activity by 9 months [28]. A meta-analysis demonstrated significantly higher rates for resolution of dactylitis and enthesitis with biologic treatment compared to placebo at both weeks 12–14 and Week 24. Interestingly, the authors found some differences in treatment response between the previous generation TNF-α inhibitors and novel biologics targeting IL-23 and IL-17 [29]. These differences may be related to differences in the study population enrolled. Regarding PALACE 1-3 trials, a consistent percentage of PsA patients had had previous treatment with biologics.

According to our data concerning patients with psoriatic arthritis with moderate activity, apremilast was still effective at improving invalidated aspects such as enthesitis and dactylitis. In multivariate analysis, the variables inversely associated with achieving low enthesitis activity were low baseline LEI. The remission of dactylitis was inversely related to baseline dactylitis score and directly by age and concomitant use of csDMARDs. The inverse association between remission and baseline activity score was also observed for peripheral arthritis (i.e., DAPSA) [30]. Data from the University of Toronto PsA clinic reported that the resolution of enthesitis was associated with lower joint activity and male sex [31]. Similar to our study, they reported that a high CRP level at baseline was associated with a better response to therapy. Therefore, patients with higher disease activity could have better outcomes [32]. Moreover, in the DANBIO registry, predictors of response to biologics in patients with PsA were a higher VAS and a CRP > 10 mg/L [33].

This study provides insights into the impact of apremilast on enthesitis and dactylitis among PsA patients in a real-life setting. In addition, this is the first Italian study to primarily focus on the outcome of dactylitis and entheseal response to apremilast in PsA. Moreover, compared to others real-life studies on enthesitis and dactylitis in PsA [19,24,25], we described a larger cohort, with a longer follow-up providing variables associated with the remission of dactylitis and enthesitis at 12 months. This study highlights, for the first time, that patients with moderate PsA treated with apremilast have a large chance of improvement in enthesitis and dactylitis involvement with a higher probability in those patients with lower baseline severity of enthesitis and dactylitis, confirming that the use of apremilast can improve some disabling aspects of psoriatic arthritis as well. 

This study has some limitations. First, the retrospective observational design leads to a possible selection bias deriving from the inclusion of patients with different probabilities of treatment response. Moreover, the relatively short follow-up period is a limitation, as patients were not analyzed beyond 12 months, even though this was longer than in other published studies. Psoriasis severity and prior treatments were not evaluated, and we do not have data regarding local injection with steroids. In a multicenter study, different factors can increase the inter-observer variability of enthesitis assessment, such as the identification of the enthesis points, the intensity of the pressure applied, the patient’s pain threshold level, or the presence of other conditions that cause pain such as fibromyalgia. All those aspects are not described in other studies. Although we did not apply the Leeds dactylitis index as a measure for dactylitis, which is a reference measure for clinical trials, we used dactylitis to estimate the score, which has been reported in a previous work [34]. Finally, even if the use of ultrasound allows for a more accurate evaluation of the treatment [35], we did not report data on sonographic screening.

These real-life data confirm that PsA patients treated with apremilast experienced a sustained improvement in enthesitis and dactylitis activity at 6 months, with further improvement achieved after 1 year and a significant rate of patients in remission. Future studies, especially in patients treated for a longer duration with apremilast, should be encouraged in order to confirm our results.

## Figures and Tables

**Table 1:** Patient baseline characteristics (all patients with enthesitis or dactylitis). Note: 47 patients are in both groups.

Baseline Characteristic		Dactylitis Subgroup	Enthesitic Subgroup
**N**		96	118
**M:F**		39:57	46:72
**Age, median (IQR) yrs**		58 (50–64)	58 (51–65)
**Smokers, n (%)**	Yes Former No Unknown	20 (20.8) 12 (12.5) 63 (65.6) 1 (1.1)	22 (18.6) 17 (14.4) 78 (66.1) 1 (0.9)
**Body Mass Index, median (IQR) kg/m^2^**		25.7 (23.4–29.0) (*)	24.9 (23.0–29.0) (**)
**PsA Duration, median (IQR), months**		44 (17–85)	42 (14–83)
**PsO Duration, median (IQR), months**		57 (19–128)	59 (15–139)
**SJC, median (IQR)**		4 (2–4)	3 (2–4)
**TJC, median (IQR)**		6 (4–10)	8 (4–12)
**LEI, median (IQR),**		-	3 (1–4)
**Dactylitis, median (IQR), fingers**		1 (1–2)	-
**CRP, median (IQR), mg/dL**		2.3 (1.0–5.0)	3.0 (1.0–7.0)
**PGA Patient (0–10), median (IQR)**		7 (6–8)	6 (5–8)
**VAS pain (0–10), median (IQR)**		7 (6–8)	7 (6–8)
**DAPSA, median (IQR)**		25.7 (20.2–33.0)	27.6 (22.6–36.0)
**Concomitant csDMARDs use, n (%)**		27 (28.1)	30 (25.4)
**Prior bDMARDs use, n (%)**		34 (35.4)	34 (28.8)
**Concomitant relevant disease, n (%)**		51 (53.1)	45 (38.1)

Data missing in 3 (*) and 8 (**) patients.

**Table 2:** Univariate and multivariate analysis of variables associated with the achievement of enthesitis remission at twelve months.

Variable	Univariate Analysis		Multivariate Analysis	
	*OR (95% CI)*	*p*	*OR (95% CI)*	*p*
Age	0.99 (0.95–1.02)	0.5		
Sex	2.34 (1.07–5.12)	0.03	2.01 (0.86–4.68)	0.11
BMI	1.11 (1.02–1.22)	0.02	1.09 (0.99–1.20)	0.09
Smoke habit	0.89 (0.33–2.40)	0.80		
PsA duration	1.00 (0.99–1.01)	0.90		
Relevant comorbidity	0.76 (0.35–1.65)	0.49		
Concomitant csDMARDs	0.97 (0.27–3.44)	0.97		
LEI, baseline	0.70 (0.54–0.91)	0.007	**0.76 (0.58–0.98)**	**0.03**

**Table 3:** Univariate and multivariate analysis of variables associated with achievement of dactylitis remission at twelve months.

Variable	Univariate Analysis		Multivariate Analysis	
	*OR (95% CI)*	*p*	*OR (95% CI)*	*p*
Age	1.04 (1.00–1.08)	0.04	**1.05 (1.00–1.09)**	**0.04**
Sex	1.18 (0.52–2.68)	0.7		
BMI	1.06 (0.97–1.16)	0.18		
Smoke habit	0.63 (0.22–1.76)	0.38		
PsA duration	0.99 (0.98–1.00)	0.15		
Relevant comorbidity	0.63 (0.28–1.43)	0.27		
Concomitant csDMARDs	2.41 (0.97–5.97)	0.06	**3.84 (1.30–11.31)**	**0.01**
Dactylitis, baseline	0.45 (0.24–0.85)	0.014	**0.41 (0.21–0.78)**	**0.007**

## Data Availability

All data generated or analyzed during the study are included in this published article.

## Supplementary Materials

The following supporting information can be downloaded at: https://www.mdpi.com/article/10.3390/jcm12123892/s1, Table S1: Patients baseline characteristics (all patients with enthesitis or dactylitis with 12 months of follow-up). Note: Forty-seven (47) patients had synchronous dactylitis and enthesitis.
