# Supplementary material for: Therapeutic Effects of Apremilast on Enthesitis and Dactylitis in Real Clinical Setting: An Italian Multicenter Study
Source: J Clin Med. 2023 Jun 7;12(12):3892. doi: 10.3390/jcm12123892 (PMC10299365; doi:10.3390/jcm12123892)
Supplement: Supplementary file 1 [file jcm-12-03892-s001.zip › jcm-2374412-supplementary.pdf]

Table 1 supplementary

Patients baseline characteristics (all patients with enthesitis or dactylitis with 12 months of follow-up). Note: Forty-seven (47) patients had synchronous dactylitis and enthesitis

**Baseline Characteristic**		**Dactylitis subgroup**	**Enthesitic subgroup**
**N**		64	66
**M:F**		39:25	27:39
**Age, median [IQR] yrs**		59 [53-65]	59 [52-65]
**Smokers, n (%)**	Yes<br>Former<br>No<br>Unknown	13 (20,3)<br>10 (15,6)<br>41 (64,1)<br>0	10 (15,2)<br>13 (19,7)<br>43 (65,1)<br>0
**Body Mass Index, median [IQR] kg/m^2**		27,1 [23,6-30,0] (*)	26,5 [23,1-29,8] (**)
**PsA Duration, median [IQR], months**		41 [15-87]	54 [17-94]
**PsO Duration, median [IQR], months**		82 [20-137]	66 [17-141]
**SJC, median [IQR]**		3 [2-4]	3 [2-4]
**TJC, median [IQR]**		5 [3-7]	6 [4-12]
**LEI, median [IQR],**		-	2 [1-4]
**Dactylitis, median [IQR], fingers**		1 [1-2]	-
**CRP, median [IQR], mg/dl**		2,2 [1,0-5,0]	3,3 [1,0-6,0]
**PGA Patient (0-10), median [IQR]**		7 [6-8]	7 [5-8]
**VAS pain (0-10), median [IQR]**		7 [6-8]	7 [6-8]
**DAPSA, median [IQR]**		24,2 [19,5-31,5]	25,6 [22,0-35,0]
**Concomitant csDMARDs use, n (%)**		22 (33,4)	17 (25,8)
**Prior bDMARDs use, n (%)**		25 (39,1)	20 (30,3)
**Concomitant relevant disease, n (%)**		37 (56,3)	28 (42,4)

Data missing in 1 (*) and 3 (**) patients
